# An innovative resident-driven mortality case review curriculum to teach and drive system-based practice improvements in the United States

**DOI:** 10.3352/jeehp.2018.15.31

**Published:** 2018-12-26

**Authors:** Nila S. Radhakrishnan, Margaret C. Lo, Rohit Bishnoi, Subhankar Samal, Robert Leverence, Eric Rosenberg, Zareen Zaidi

**Affiliations:** 1 Department of Medicine, University of Florida College of Medicine, Gainesville, FL, USA; 2 Department of Medicine, University of Texas Health Science Center San Antonio, San Antonio, TX, USA; Hallym University, Korea

**Keywords:** Mortality reduction, Morbidity and mortality conference, Internal medicine resident education, Systems-based practice, United States

## Abstract

**Purpose:**

Traditionally, the morbidity and mortality conference (M&MC) is a forum where possible medical errors are discussed. Although M&MCs can facilitate identification of opportunities for systemwide improvements, few studies have described their use for this purpose, particularly in residency training programs. This paper describes the use of M&MC case review as a quality improvement activity that teaches system-based practice and can engage residents in improving systems of care.

**Methods:**

Internal medicine residents at a tertiary care academic medical center reviewed 347 consecutive mortalities from March 2014 to September 2017. The residents used case review worksheets to categorize and track causes of mortality, and then debriefed with a faculty member. Selected cases were then presented at a larger interdepartmental meeting and action items were implemented. Descriptive statistics and thematic analysis were used to analyze the results.

**Results:**

The residents identified a possible diagnostic mismatch at some point from admission to death in 54.5% of cases (n= 189) and a possible need for improved management in 48.0% of cases. Three possible management failure themes were identified, including failure to plan, failure to communicate, and failure to rescue, which accounted for 21.9%, 10.7 %, and 10.1% of cases, respectively. Following these reviews, quality improvement initiatives proposed by residents led to system-based changes.

**Conclusion:**

A resident-driven mortality review curriculum can lead to improvements in systems of care. This novel type of curriculum can be used to teach system-based practice. The recruitment of teaching faculty with expertise in quality improvement and mortality case analyses is essential for such a project.

## Introduction

The morbidity and mortality conference (M&MC) is traditionally utilized in academic medical centers as a forum to identify and discuss adverse events. Despite its long-standing tradition, the M&MC has 2 main flaws. First, the considerable variation in the structure of these conferences means that there is little consensus on the goal, method, and format [1]. Second, the literature regards the M&MC as a crude means of addressing errors since many adverse events are identified through other processes and are often not reported in M&MCs [2,3]. M&MCs have been further criticized for being a public forum for blame allocation [4]. Many institutions have restructured their M&MC format to identify and implement improvement initiatives and to sharpen a focus on mortality reduction through system improvements [5,6,7]. Nonetheless, the literature indicates that M&MCs rarely lead to system-based actions [1,8,9]. A survey of US internal medicine residency programs revealed that most programs contained MM&Cs as a traditional forum to discuss medical errors and pathophysiology related to adverse events and deaths. However, only half of the residency programs had established a process for handling and following up on the errors identified in these conferences [1]. The Accreditation Council for Graduate Medical Education (ACGME) mandates as a core requirement that residents participate in quality improvement projects and ‘understand common principles and techniques of quality improvement’ [10]. Participation in clinical case review and peer review is a fundamental component of the contributions made by physicians-in-training to quality improvement.

Although research has investigated how to involve residents in patient safety and peer review through the M&MC format, few studies have described the process of engaging residents in real-time patient case reviews focused on mortality. This paper describes the process of planning and implementing a resident-driven mortality case review curriculum to teach residents how to critically analyze factors associated with in-hospital mortality and to increase engagement in quality improvement initiatives related to mortality prevention. We also report results regarding the nature of potential system errors identified by residents and the actions taken by the department of medicine in response.

## Methods

### Ethical statement

The Institutional Review Board of University of Florida approved this study as a quality improvement project (QIPR Project ID 182).

After IRB approval to study the curriculum, (IRB201701579) the learners were surveyed about the curriculum anonymously and with informed consent.

### Educational setting and participants

In 2010, to understand factors associated with preventable mortality, an inter-professional team of physicians, pharmacists, nurses, and quality analysts at the University of Florida Health Hospital adapted the Kaiser Permanente Mortality Review Tool to perform a pilot review of 50 inpatient deaths among patients with advanced illness [11,12]. The review sought to answer 3 questions: (1) Was there evidence of failure to rescue the patient?; (2) Was there evidence of failure to create contingency plans for patient decompensation?; and (3) Was there evidence of failure to communicate among the practitioners caring for the patient? Although most deaths occurred as a consequence of underlying progressive deterioration of comorbid conditions, some deaths were potentially preventable due to failure of rescue (i.e., failure of early transfer to the intensive care unit or to perform other escalations of diagnostic and therapeutic interventions). Prior studies noted that cases of failure to rescue can result from diagnostic errors, failure to coordinate care among physician specialties, and communication breakdowns [13]. Early warning systems were subsequently implemented and the department of medicine M&MC was restructured to focus on these cases. While our medicine residents attended these M&MCs, their experiential training on patient safety was limited to a 1-month quality improvement/patient safety curriculum modeled on the Institute of Health Improvement (IHI) Open School during their yearly ambulatory rotation. In our 2015 program survey, only 83% of medicine residents felt that they had participated in quality improvement activities. In response, departmental experts in patient safety, along with the residency program directors, designed and implemented a monthly resident-driven mortality case review curriculum. This curriculum takes place during the ambulatory rotation of the University of Florida Internal Medicine Residency Program in Gainesville, Florida.

### Materials and subjects

Based on the above logic, participants reviewing cases were participating in a quality review project, and this was clearly stated prior to case review. We utilized the ‘IHI Move Your Dot’ framework to plan the new format of our M&MC and to design our mortality case review curriculum. This framework helps to inform a hospital’s organizational performance related to mortality. The key principle of this framework involves case-based analysis to identify local system issues that can be improved [6,7].

### Technical information

**Fig. 1.**
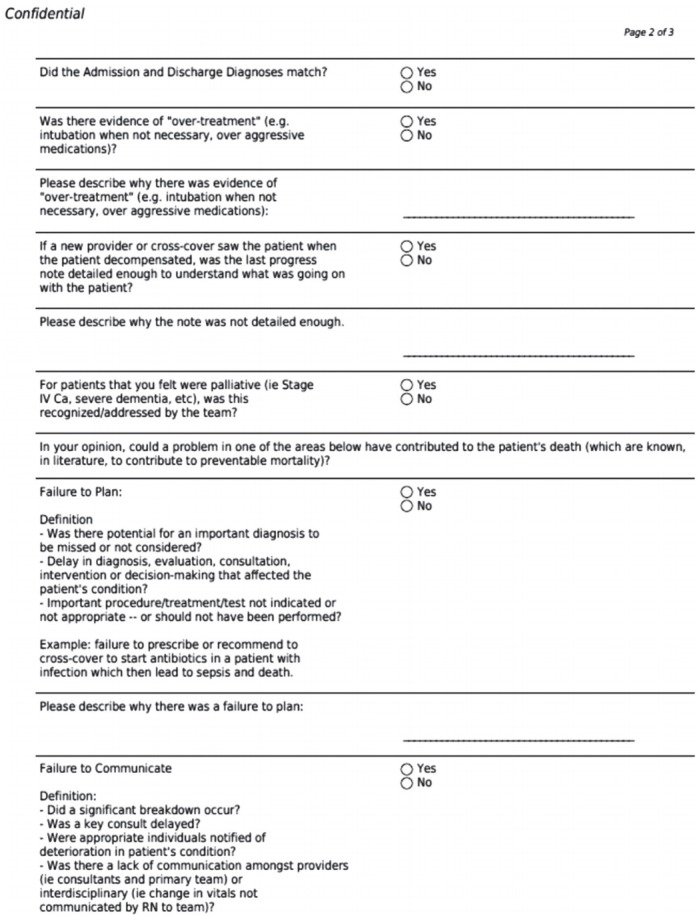
Mortality review worksheet.

Development of the case review worksheet: Using the Move Your Dot framework, residents used a case review worksheet to categorize and track mortality cases into categories of management failure that were identified during case reviews (Fig. 1). Longitudinal data from each worksheet were tracked in the online HIPPA-compliant Research Electronic Data Capture (REDCap) platform.

**Fig. 2.**
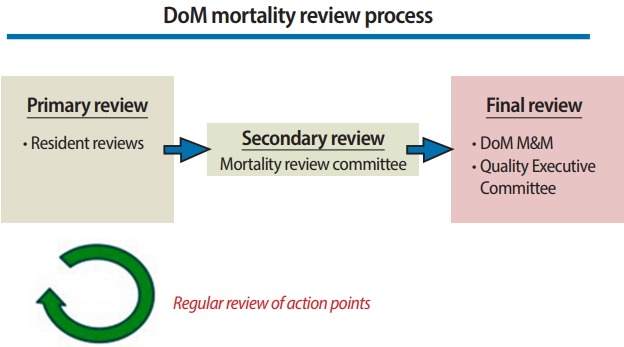
DoM mortality review process. DoM, department of medicine.

**Fig. 3.**
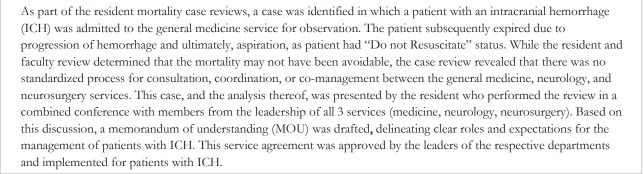
Example of a mortality event and actions generated by the resident-led team.

Mortality case review process and sessions: Cases were selected to include every instance of mortality from the general medicine teaching service. Residents reviewed every mortality case from the medicine teaching services and identified areas for improvement in diagnosis and management using the mortality worksheet during their independent review of the cases. Residents then met with a faculty mentor to discuss the cases and to analyze potential problems in medical management that impacted mortality risk. The debriefing session consisted of a brief case presentation followed by a discussion of whether the cases involved any of the categories of failure that had been noted. There was also a discussion of the categories of failure. During these debriefing sessions, residents proposed potential action items to prevent similar mortality issues. Mortality cases in need of further review were subsequently forwarded to an executive committee or interdepartmental conference specifically organized to discuss the case and to develop action items for improvement (Fig. 2). A synthesis of these in-depth discussions and analyses of mortality cases led to a consensus regarding action items to implement to address systemwide issues. Fig. 3 presents an actual example from the conference and actions taken.

### Statistics and data analysis

Case review data on mortalities from the medicine teaching services were analyzed. Descriptive statistics were used to analyze the numeric data regarding mortality and management failure categories. To organize and index the data set, 2 of the authors (NSR and ZZ) initially coded the data. The Braun and Clarke [14] framework of qualitative thematic analysis was used to analyze the open-ended responses. Following the 6 phases described by Braun and Clarke [14], we independently analyzed the data and identified management failure themes, focusing on patterns and richness of responses rather than the number of responses, and assigned comments to themes. Another author peer-reviewed the themes for accuracy and an audit trail was maintained with comments.

## Results

**Fig. 4.**
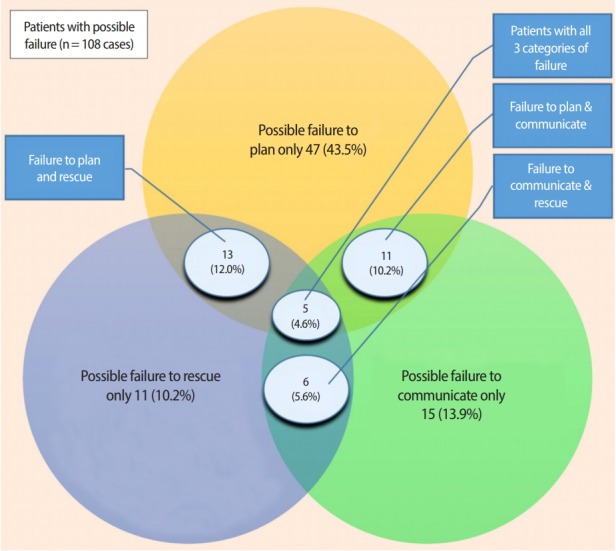
Overlap of failure categories in the 108 cases with a possible failure.

From March 2014 to September 2017, 347 consecutive mortality cases were reviewed by 107 internal medicine residents. The residents identified a possible diagnostic mismatch at some point from admission to death in 54.5% of cases (n= 189) and a possible need for improved management in 48.1% of cases (n= 167) (Table 1). Three possible management failure themes were identified, including failure to plan, failure to communicate, and failure to rescue, which were implicated in 21,9% (n= 76), 10.7% (n= 35), and 10.1% of cases (n= 35), respectively. Three additional themes were identified including possible overtreatment (9%, n= 32), documentation issues (12%, n= 41), and lack of involvement of the palliative care team (19%, n= 67). The categories of possible failures were not mutually exclusive and many of the cases had multiple categories that overlapped. A total of 108 patients had 1 or more possible failures (31.1%). Of these 108 cases, 73 (21.0%) had a single category of failure and 35 (10.1%) had more than 1 category of failure. Eleven cases (10.2%) had possible failures to plan and to communicate. Six cases (5.6%) had possible failure to communicate and to rescue. Thirteen cases (12.0%) had possible failures to plan and to rescue. In 5 cases (4.6%), all 3 categories of possible failure were identified (Fig. 4). The raw data are available in Supplement 1.

For theme 1, failure to plan, 2 sub-themes were identified, relating to possible delays in diagnosis and delays in treatment. The delay in diagnosis sub-theme identified by residents included possible delays in the diagnosis of evolving life-threatening conditions (e.g., septicemia, acute myocardial infarction, or stroke) and delays in ordering diagnostic tests such as computed tomography (CT) scans or lumbar punctures. Exemplary comments include:

“Possible delay in diagnosis regarding the patient becoming septic. She was hypotensive.” (R01)
“I think he may have benefited from early lumbar puncture on the day of his admission.” (R02)
“Patient didn’t get CT abdomen when he was becoming febrile abdominal pain and distention noted by consult note day 2, CT scan was done day 4, possible 2 day delay.” (R03)

A delay in treatment was identified in cases where additional treatment actions were needed but failed to occur:

“Service was consulted for catheter placement was consulted for dialysis catheter placement however was unable to do the procedure on the day planned. The morning of patient’s decompensation as per consult note there was significant respiratory decline. An attempt at placing a catheter by bedside by primary team, higher level of care may have addressed volume overload earlier.” (R04)
“The patient developed marked respiratory distress after eating breakfast. Patient was given nebulizers and steroids however continued to be tachypneic in 130s–140s. This may be aspiration however patient had recent surgery and essentially immobile thus high risk of deep vein thrombosis/pulmonary embolism…. Notes report ‘antibiotics for aspiration’ however none appeared ordered. Heparin may not have been enough for her respiratory failure.” (R05)

Theme 2, failure to communicate, referred to breakdowns in communication among physicians, nurses, and family members. Examples include:

“Consult team had multiple bedside discussions regarding code status without primary team coordination and changed code status, documented frustration with primary team’s involvement in code status discussions and documented frustrations over realizing solumedrol and granulocyte-colony stimulating factor had been ‘held.’ Not clear that these recommendations or frustrations were addressed directly with the team. No documentation of team meetings.” (R06)
“It is difficult to determine how this was discussed, but nursing staff sent a text page at approximately 7 PM on day prior to death concerned about Modified Early Warning Score. Unknown if this was followed up.” (R07)
“Family and patient not informed of severity of condition on admission (or at least no documentation exists to state they were informed.” (R08)

Theme 3, possible failure to rescue, had 2 sub-themes, relating to delays in the recognition of deteriorating vital signs and inappropriate responses to changes in patients’ clinical condition.

“Tachycardia not addressed entire hospitalization. Severe abdominal/chest pain on HD#2... Although CT performed, may have missed underlying gastric perforation/ischemia which was noted on esophagogastroduodenoscopy HD#4.” (R03)
“Patient had a SWAT event with hypotension and syncope the day prior to decompensation and death. Twenty-four hours prior to death, the patient was found to have oliguria and lactic acidosis, but was not transferred to higher level of care. It is likely that the patient’s blood pressure medication of metoprolol was blunting tachycardia response which may have warned the team.” (R07)
“Patient noted to be tachycardic to 130s and starting to become more hypotensive in the emergency department around 4 AM, no fluids given until 8 AM. At 8 AM patient is given 500 mL normal saline (NS) by day team, however per chart continues to be hypotensive with systolic blood pressure 70s–80 and mean arterial pressures 46–50s. No further fluids given or action taken until approximately 1:30 PM when another L NS given.” (R08)

## Discussion

Herein, we describe the educational value of our innovative mortality case review curriculum to teach residents and drive systembased improvements for mortality reduction. Through facilitated reviews of real-life cases of mortality, residents were able to identify management failure themes. Approximately 50% of cases involved possible failure to plan care, communication issues with team members, and failure to rescue the patient prior to a terminal event. A ripple effect of the residents’ critical analyses of real-time decisions involved in these cases of mortality was the proposal of quality improvement initiatives leading to system-based changes.

Our curriculum contributes to the current literature demonstrating that residents can be successfully involved throughout the quality process. Tad-Y et al. [5] described a process for a system-based M&MC with interprofessional and interdisciplinary participation that led to action items. Trainees attending the conference completed a modified fishbone diagram. A core team debriefed after the M&MC conference and followed up on suggestions and actionable items. Kwok et al., described a hospital-wide structured M&MC, with representation from ‘high-impact’ clinician groups and ‘core specialty’ representation [15]. Our curriculum is unique in that the residents were active participants in critically reviewing actual patient charts, documenting their findings regarding diagnostic errors, and proposing potential action plans. They then advanced to interdisciplinary teambased debriefings of the mortality cases including pharmacy, nursing, and subspecialty physicians from various departments. Examples of the action items created include a protocol for management of intracranial hemorrhage (Table 2), a process for early involvement of the palliative medicine team with terminally ill patients, and a protocol for early sepsis alerts. Since we found physician-to-physician and nurse-to-physician communication possible failures, we introduced a HIPAA-compliant secure text messaging application for smartphones.

**Fig. 5.**
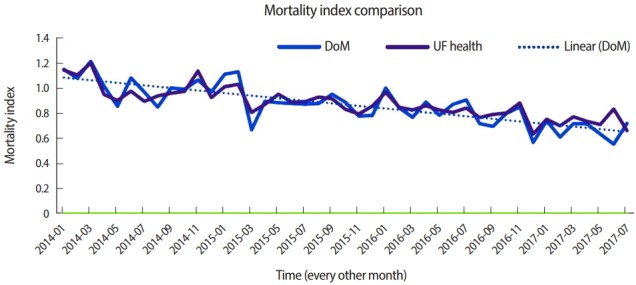
The mortality index (observed/expected [University Health Consortium/Vizient]) for the DoM. DoM, department of medicine; UF, University of Florida.

During the implementation of our new curriculum, the mortality index (observed/expected [University Health Consortium/Vizient]) for the department of medicine improved from 1.21 to 0.7 (Fig. 5). However, as multiple other quality initiatives were undertaken in the hospital system, we cannot be sure to what extent the action items generated from the mortality reviews resulted in the decrease of our mortality index-enhanced.

A limitation of our curriculum was the lack of resident continuity from the initial case review to the proposal of action items and final system-based implementation. This limitation was unavoidable due to the residents’ schedule and the lag time needed to implement system-based changes. Future directions may include an electronic learning management system that could be used to track which case was discussed and allow for direct feedback and discussion on a message board. This would obviate the need for the residents’ physical presence to receive feedback. Given the experiential teaching focus of our curriculum, we did not directly assess the impact of the curriculum on residents’ patient safety education (i.e., residents’ perception or knowledge improvement on the M&MC process and principles). Nevertheless, our curriculum applied adult learning principles for residents to critically analyze and identify mortality causes and engage in change management.

The recruitment of teaching faculty with expertise in quality improvement and mortality case analyses is essential for such a project. A key lesson we learned was the importance of identifying and recruiting a small group of core faculty with expertise in quality improvement and a strong interest in teaching learners. We also emphasize the importance of early buy-in and close partnership with the residency program directors and hospital administration. To implement a similar mortality case review curriculum and M&MC redesign, we recommend early involvement and buy-in from the Office of Graduate Education, the Departmental Quality Improvement leaders, and the Senior Dean for Clinical Affairs.

Future directions for this curriculum include having resident teams, ideally with representation from each post-graduate year, work on longitudinal action items. Each team should have a faculty mentor and designate ‘resident quality champions’ to work on action items identified through case reviews. These quality teams of faculty, residents, and interprofessional representatives could conduct longer quality projects.

In conclusion, our resident-led mortality case review curriculum not only satisfied the ACGME’s requirement of experiential quality training for residents, but also promoted system-based improvements within the institution. We encourage residency programs to build similar curricula and have mechanisms in place to engage residents in the institutional mortality review process.

## Figures and Tables

**Table 1.** Deficiency categories

Deficiency categories	No. (%)
Possible failure to plan	76 (22)
Possible failure to communicate	37 (11)
Possible failure to rescue	35 (10)
Possible over-treatment	32 (9)
Documentation issues	41 (12)
Palliative care not involved	67 (19)
One or more issues	167 (48)

**Table 2.** Thematic analysis of categories of failure

Theme	Sub-themes
Failure to plan	Delays in diagnosis
	Delays in treatment
Failure to communicate	Physician-to-physician communication
	Nurse-physician communication
	Patient or family communication
Failure to rescue	Delay in recognition of changes in vital signs
	Delay in responding to changes

## Supplementary materials

Supplement 1. Raw data. Data files are available from https://doi.org/10.7910/DVN/FABH69

Supplement 2. Audio recording of the abstract.
